# Supporting the Transition From Hospital to Home for Older Adults: Case Study Results

**DOI:** 10.1093/geroni/igaa057.266

**Published:** 2020-12-16

**Authors:** Lori Weeks, Brittany Barber, Eileigh Storey MacDougall, Elaine Moody, Lexi Steeves-Dorey, Grace Warner, Marilyn Macdonald, Ruth Martin Misener

**Affiliations:** 1 Dalhousie University, Halifax, Nova Scotia, Canada; 2 Nova Scotia Health Authority, Halifax, Nova Scotia, Canada

## Abstract

It is often during transitions between health services that many issues arise for older adults, such as care that is poorly coordinated, additional burden placed on family and friend caregivers, and inappropriate placement in nursing homes. The Home Again program delivered by the provincial health authority in Nova Scotia, Canada, provides transitional care through providing additional support beyond what is normally provided through home care services to help older adults transition home after a hospital admission. The purpose of this research was to identify what factors contribute to older adults being placed unnecessarily in a nursing home when they could receive care through the Home Again program. Through using a retrospective multiple case study design, we analyzed interviews for five cases including older adult patients, their family or friend caregivers, and healthcare professionals in each case. Results indicate all hospitalized patients experienced a major health event or rapidly declining health. All family and friend caregivers experienced burnout and frustration from the lack of sufficient home care supports and quality of services available, such as services provided throughout the night. Healthcare professionals discussed that patients were placed on a waiting list for nursing homes due to lack of home care supports and resources for caregivers. This study contributes to our knowledge about better processes to ensure that hospitalized older adults are not unnecessarily admitted to nursing homes which can result in reduced healthcare costs and improved delivery and quality of care to older adults and their family and friend caregivers.

